# Establishing Infection Prevention and Control Centers of Excellence in Ethiopian Hospitals

**DOI:** 10.1017/ash.2026.10636

**Published:** 2026-06-23

**Authors:** Karen Wu, Janelle Franklin, Selamawit Weledgebreal, Beniam Feleke Welebo, Alemu Derseh, Kasu Tola Bifa, Hailegebriel Abomsa Bedane, Fekadu Nigussie, Carolyn Herzig, Deneke Ayele, Markos Paulos, Abas Hassen Yesuf, Getachew Kassa, Tamrat Assefa, Berhanu Tekle, Zenebe Melaku

**Affiliations:** 1 CDC; 2 Centers for Disease Control and Prevention; 3 US-CDC in Ethiopia; 4 CDC Ethiopia; 5 Resolve to Save Lives; 6 CDC Foundation; 7 Division of Healthcare Quality Promotion, Centers for Disease Control and Prevention; 8 Ministry of Health Ethiopia; 9 ICAP at Columbia University; 10 ICAP @Columbia University; 11 ICAP @ Comlubia University; 12 ICAP in Ethiopia

## Abstract

**Background** Ethiopia’s Ministry of Health (MoH), in collaboration with ICAP at Columbia University and the U.S. Centers for Disease Control and Prevention, sought to establish five infection prevention and control (IPC) Centers of Excellence (CoEs) with the ultimate goal of promoting effective and sustained implementation of IPC interventions. The CoEs are based on MoH IPC CoE guidance and leverage capacity gained through previous initiatives including implementation of a national IPC Advanced Training Program and a national monitoring and evaluation system. CoEs are encouraged to develop innovative solutions to challenges and serve as a reference for other healthcare facilities. We describe the initial phase of establishing IPC CoEs. Methods In 2023, five hospitals were selected based on the following criteria: experience implementing IPC interventions, hospital size and location, supportive leadership, and willingness to disseminate findings. Selected hospitals used the WHO multimodal improvement strategy to strengthen IPC over nine months, starting with environmental cleaning programs in maternity units. Facility-level baseline assessments and unit-level environmental cleaning needs assessments were created and conducted, followed by the development of operational plans to address identified gaps. The implementation team provided training, mentorship, and resources (e.g., CDC’s Best Practices for Environmental Cleaning in Healthcare Facilities) to each facility. Initial and follow-up audits assessing visual cleanliness and availability of appropriate cleaning materials were conducted after <3 months to assess progress. Results Baseline assessment demonstrated that all hospitals outsourced environmental cleaning services. None of the hospitals provided regular training upon employment or had job-aids for environmental cleaning staff. One hospital had an environmental cleaning policy. The maternity unit needs assessment identified gaps in IPC standard operating procedures (SOPs) at four hospitals. During implementation, all facilities developed operational plans to guide improvements, subsequently creating or updating environmental cleaning policies and SOPs. Hospitals worked with environmental cleaning companies to train cleaning staff and monitor cleaning. Mean audit scores across four hospitals increased from 51% at baseline (range 22-90%) to 83% (range 71-94%); a follow-up audit was not conducted at one hospital. Conclusion Initial efforts to establish IPC CoEs demonstrated that hospitals with existing IPC capacity built from national programs successfully led interventions to address identified gaps in environmental cleaning programs. Further work is needed to determine whether the interventions are sustainable long-term and whether interventions can be applied to other wards and healthcare facilities in the country.

